# The host immune response of a discharged COVID-19 patient with twice reemergence of SARS-CoV-2: a case report

**DOI:** 10.1186/s12879-021-06679-3

**Published:** 2021-09-23

**Authors:** Xiaobin Zao, Yang Zhou, Yijun Liang, Xu Cao, Hening Chen, Xiaoke Li, Yong’an Ye

**Affiliations:** 1 Dongzhimen Hospital, Beijing University of Chinese Medicine, 5 Haiyuncang Rd, Dongcheng District, Beijing, 100700 People’s Republic of China; 2 Key Laboratory of Chinese Internal Medicine of Ministry of Education and Beijing, Dongzhimen Hospital, Beijing University of Chinese Medicine, 5 Haiyuncang Rd, Dongcheng District, Beijing, 100700 People’s Republic of China; 3 Beijing Ditan Hospital, Capital Medical University, 8 Jingshun East Street, Chaoyang District, Beijing, 100015 People’s Republic of China; 4 Institute of Liver Diseases, Beijing University of Chinese Medicine, 5 Haiyuncang Rd, Dongcheng District, Beijing, 100700 People’s Republic of China

**Keywords:** SARS-CoV-2, COVID-19, Reemergence, RNA test, Host immune response

## Abstract

**Background:**

The coronavirus disease 2019 (COVID-19) caused by severe acute respiratory syndrome coronavirus 2 (SARS-CoV-2) has become a global pandemic. There have been reports that long-term SARS-CoV-2 RNA shedding and re-infection of COVID-19 patients existed. However, the specific mechanism, diagnosis, and treatment of COVID-19 are still unclarified.

**Case presentation:**

In this case, we reported a 64-year-old patient who had a long-term course of COVID-19 for 174 days with two retests of SARS-CoV-2 RNA positive after discharging from the hospital. The patient’s serum immunoglobulin G (IgG) of SARS-CoV-2 tested positive after the initial infection. And during treatment, the CD4 + T cell count and ratio to peripheral blood mononuclear cell (PBMC) were in dynamic change.

**Conclusions:**

Our results suggested that the host immune system responded with IgG production after SARS-CoV-2 infection, but was not protective enough for the patient. The reemergence of SARS-CoV-2 could be related to the cell count and proportion of CD4 + T cells in PBMC. And the increase of CD4 + T cells after treatment may help to clear the virus.

## Background

The quantitative real-time polymerase chain reaction (qRT-PCR) was used generally to detect severe acute respiratory syndrome coronavirus 2 (SARS-CoV-2) with samples of the nasopharynx, pharynx, bronchoalveolar lavage fluid, feces, and blood [1]. And there has been a patient with long-term SARS-CoV-2 RNA shedding for up to 105 days after initial diagnosis as coronavirus disease 2019 (COVID-19) [2]. Additionally, there were some symptomatic and asymptomatic cases who had RNA test positive again for SARS-CoV-2 following a period of negative testing exited [3]. Thus, it’s been a hotspot that whether it is long term viral persistence, sampling issues, or re-infection for ‘recovered’ patients with re-tested SARS-CoV-2 RNA positive [4–6]. However, the exact mechanism for SARS-CoV-2 reemergence is still unclear, and high-level vigilance remains required for this kind of reemergence. Here, we reported a case of COVID-19, who was twice-time reemergence and had a long-term course of SARS-CoV-2 for 174 days.

## Case presentation

**Fig. 1:**
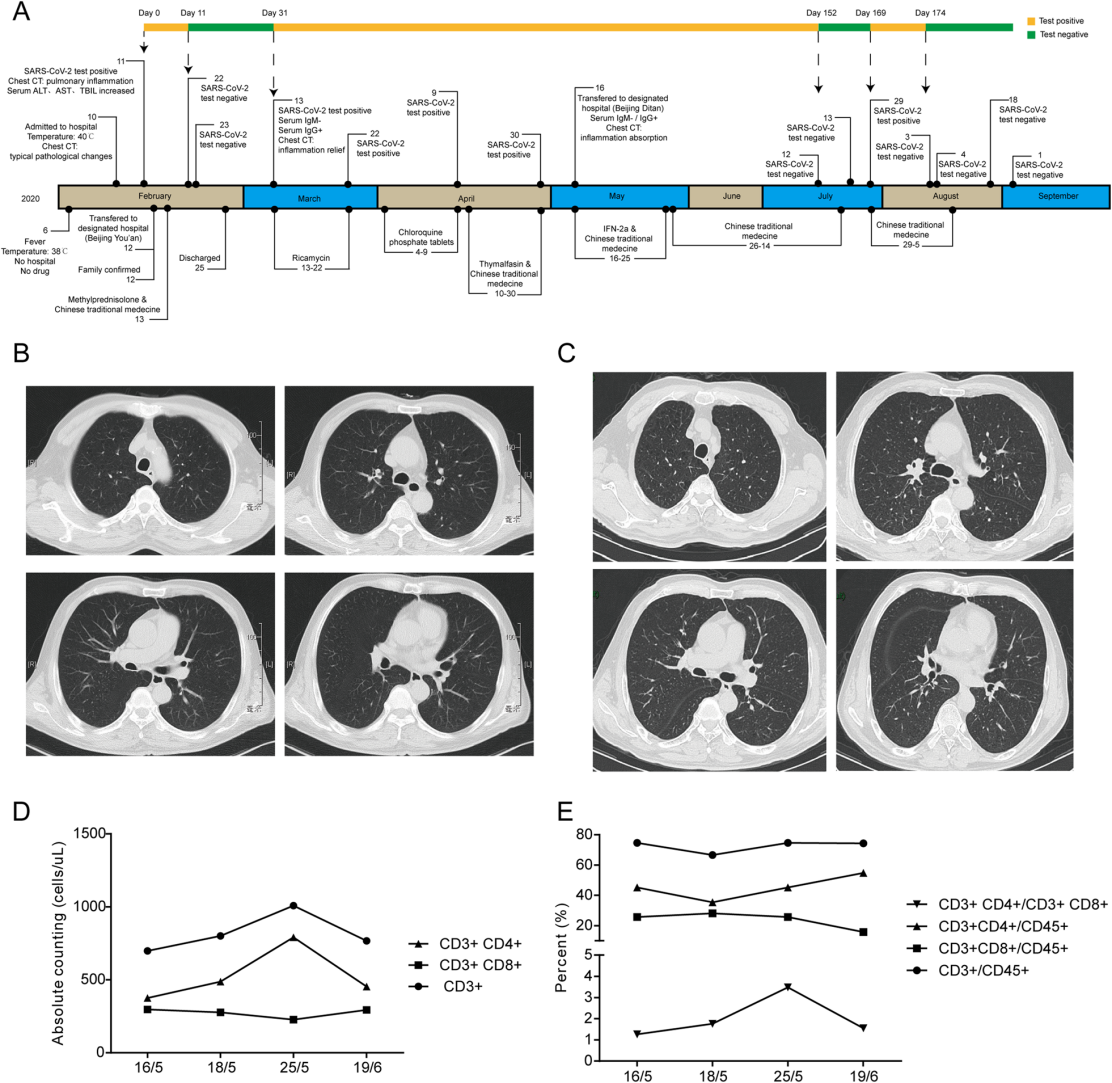
The time axis and examination results of the patient. **A** From the first visit to the hospital to the final discharge, the patient's condition, tests, and medication are recorded. The yellow marker represents a positive SARS-CoV-2 RNA test period, and the green marker represents a negative test period according the diagnose criteria; **B** The representative chest CT scan images of the patient on February 10th; **C** The representative chest CT scan images of the patient on May 16th; **D** The cell counts of T cells in the patient’s PBMC; **E** The cell percent of T cells in the patient’s PBMC.

The patient was a 64-year-old Chinese man who had baseline comorbidities including coronary atherosclerotic heart disease, high blood pressure, and diabetes. The epidemiological history of the patient: no history of travel in Wuhan and surrounding areas, the patient's son-in-law and the mother of the patient's son-in-law were both diagnosed with COVID-19 on February 12, 2020. The medications he takes daily include Aspirin, Atorvastatin, Bisoprolol, and Acarbose. As shown in the time axis of Fig. 1A, he developed a fever up to 38 ℃ on February 6th with a dry cough and mild fatigue but had no treatment. Four days later, he developed a more severe dry cough and his body temperature rose to 40 ℃, and then he went to the Peking University Third Hospital. The chest computed tomography (CT) scan showed a symmetrical thoracic cage, centered trachea, no displacement of the mediastinum, and multiple ground-glass opacity under the pleura of both lungs (Fig. 1B). The sera alanine aminotransferase (ALT), aspartate aminotransferase (AST), and total bilirubin (TBIL) were slightly increased. The following nasal swab sample of the SARS-CoV-2 test was positive with the qRT-PCR assay targeting ORF1ab and N genes of the virus genome (day 0). The patient was then transferred to the Beijing Youan hospital.

At admission, the patient’s physical condition was examined and, the oxygen saturation (SaO2%) was 95%-98%. And the CT result showed severe inflammation in his lungs. Then, the patient received treatment including Methylprednisolone (40 mg, day 2), Bicyclol piece, and Silybin glutamine tablets for liver protection. After treatment, the patient's liver enzymes returned to normal, cough and other symptoms gradually decreased. The qRT-PCR examination for SARS-CoV-2 of the patient was performed on days 11 and 12, and both tests were negative. Since the discharge criteria of the COVID-19 is two consecutive negative SARS-CoV-2 RNA tests, the patient was then discharged from the hospital and was instructed to receive a quarantine at home. Besides, there was no new infection of SARS-CoV-2 confirmed among his family and close contact.

First-time reemergence: On day 31, the patient was tested positive for SARS-CoV-2 when he returned to the hospital for a follow-up. He complained of no symptoms, including fever, cough, sputum, chest tightness, and dysphoria. However, the serum immunoglobulin G (IgG) of SARS-CoV-2 was positive, and the serum immunoglobulin M (IgM) of SARS-CoV-2 was negative. These results suggesting that the adaptive immune responded after the first infection, and this temporal reemergence might not be due to re-infection but re-activation of the virus [7]. A chest CT scan of the patient on day 31 showed grinding glass shadow of both lungs was absorbed more than before. Subsequently, the patient was treated with Chloroquine phosphate tablet (500 mg, twice a day, day 52–59). However, upon completion of the treatment, the patient still failed to meet the discharge criteria. The patient was then prescribed Thymalfasin (1.6 mg, hypodermic injection, day 59–69) for injection of the next 20 days. During this stage of treatment, the patient took traditional Chinese medicine (TCM) decoction, which consisted mainly of Honeysuckle, Forsythia, Scutellaria, Fritillary mother, and Almond, to clear heat and detoxify intermittently. After treatment, the patient still did not meet the discharge criteria. On May 16th, 2020, the patient was transferred to Beijing Ditan Hospital for further treatment.

A repeat chest CT examination of the patient on May 17th, 2020, revealed clear veins and diffuse balloon cavities in both lungs, and inflammation in the lungs was absorbed (Fig. 1C). The patient had no noticeable abnormalities in liver function, kidney function, electrolytes, and blood glucose. Meanwhile, the serum IgM of SARS-CoV-2 was turned negative. Hereupon, IFN-2a was administered for 10 days (5 MIU daily, aerosol inhalation, day 95–105). And the TCM therapy was used for clearing heat and removing dampness detoxification (days 105–154) in consideration of the patient’s abdominal distension, poor sleep at night, unformed stool, dark red tongue, fat, thin white greasy coating, and slippery string pulse. During this treatment stage, cell counts of peripheral blood lymphocytes were performed and recorded. The results showed that after treatment, the number (Fig. 1D) and ratio (Fig. 1E) of CD4 + T cells in PBMC were both upregulated. On July 12th, 2020, and July 13th, 2020, the patient was tested negative for SARS-CoV-2 RNA two consecutive times, met the discharge criteria, and was discharged on July 14th, 2020. At this point, 152 days had passed since the patient's onset of the COVID-19 disease.

Second reemergence: On July 28th, 2020, two weeks after being discharged from the hospital, the patient was found SARS-CoV-2 RNA amplification in a reexamination. On July 29th, 2020, his sample was collected again, and the SARS-CoV-2 RNA test was positive. The patient was hospitalized again for a second reemergence. This time, he had no fever, cough, sputum, or any other symptoms of admission. His chest CT showed no increased hilar shadow of the two lungs and no inflammatory manifestation. During this period, the patient had no obvious symptoms, but the SARS-CoV-2 RNA tests were still positive. Thus, at that stage, the patient received TCM treatment which was mainly used to improve immunity and help to clear the virus. The main components of this TCM formula were: Ginseng, Bupleurum, Radix scutellariae, Angelica, Radix achyranthes, Honeysuckle. After the treatment, the SARS-CoV-2 RNA tests of the patient were negative on days 174 and 175, where he met the discharge criteria and was discharged on August 5th, 2020. The patient continued to take TCM treatment after discharge. Two weeks and four weeks after discharge, the patient was re-examined for SARS-CoV-2 RNA, which were both negative. The detailed SARS-CoV-2 RNA test results of the patient were in Table 1.

**Table 1:** The SARS-CoV-2 RNA tests of the patient from 2/11/2020 to 8/3/2020

Date	Test	Date	Test	Date	Test	Date	Test	Date	Test	Date	Test	Date	Test
2/11	+	3/13	+	4/9	+	5/16	+	6/1	+	7/1	+	8/2	−
2/22	−	3/22	+	4/30	+	5/17	+	6/2	+	7/2	−	8/3	−
2/23	−					5/18	+	6/3	+	7/3	+		
						5/19	+	6/4	+	7/4	+		
						5/20	+	6/5	+	7/6	−		
						5/21	+	6/6	+	7/7	+		
						5/22	+	6/7	+	7/8	−		
						5/23	+	6/8	+	7/9	+		
						5/24	+	6/9	+	7/11	+		
						5/25	+	6/10	+	7/12	−		
						5/26	+	6/11	+	7/13	−		
						5/27	+	6/12	+	7/28	+		
						5/28	+	6/13	+	7/29	+		
						5/29	+	6/16	−	7/30	+		
						5/30	+	6/18	+	8/2	−		
						5/31	+	6/19	+	8/3	−		
								6/21	−				
								6/22	+				
								6/24	−				
								6/25	+				
								6/26	−				
								6/27	+				
								6/28	−				
								6/29	+				

+ , test positive; −, test negative

## Discussion and conclusions

Like previous reports, our observations also offered evidence for long-term shedding of SARS-CoV-2. The viral load result in a sample may fluctuate and be restricted by testing methods [8], where false-negative results occur during the disease process. On the other side, temporal but inadequate immune response and drug intervention could temporarily suppress the virus; however, once the drug is discontinued or immune function compromised, the re-activation of SARS-CoV-2 occurs. It is not clear whether this positive finding is associated with disease transmission potential. Thus, giving the disastrous consequences of transmission, a prolonged observation and multiple dimensions of virological assessment should be considered for such patients.

Furthermore, the study showed that the balance between regulatory and cytotoxic SARS-CoV-2-reactive CD4 + T cells in COVID-19 patients was broken, and a reduced proportion of regulatory CD4 + T cells was observed [9]. And the T-cell subset counts including CD8 + and CD4 + T cells in peripheral blood could be used as discriminatory biomarkers for diagnosis and severity prediction of COVID-19 [10]. Our results further suggested that the reemergence of SARS-CoV-2 could be related to the cell count and proportion of CD4 + T cells, and the increase of CD4 + T cells after treatment may help the host immune to clear the viral infection. What is noteworthy is that the researchers found that six months after infection, the neutralizing antibodies in COVID-19 patients were significantly lower than during the acute phase, which increased the risk of re-infection for patients presenting compatible symptoms of COVID-19 [11]. In our report, the patient had recurrent COVID-19 while his serum-IgG was positive, indicating that a low antibody level may not provide sufficient protection against SARS-CoV-2 re-infection. However, it needs more evidence to prove.

## Data Availability

Not applicable (no datasets were generated or analyzed during the current report).

## Abbreviations

COVID-19: Coronavirus disease 2019
SARS-CoV-2: Severe acute respiratory syndrome coronavirus 2
qRT-PCR: Quantitative real-time polymerase chain reaction
CT: Computed tomography
ALT: Alanine aminotransferase
AST: Aspartate aminotransferase
TBIL: Total bilirubin
PBMC: Peripheral blood mononuclear cell
IgG: Immunoglobulin G
IgM: Immunoglobulin M
